# Computing and Applying Atomic Regulons to Understand Gene Expression and Regulation

**DOI:** 10.3389/fmicb.2016.01819

**Published:** 2016-11-24

**Authors:** José P. Faria, James J. Davis, Janaka N. Edirisinghe, Ronald C. Taylor, Pamela Weisenhorn, Robert D. Olson, Rick L. Stevens, Miguel Rocha, Isabel Rocha, Aaron A. Best, Matthew DeJongh, Nathan L. Tintle, Bruce Parrello, Ross Overbeek, Christopher S. Henry

**Affiliations:** ^1^Computation Institute, University of ChicagoChicago, IL, USA; ^2^Computing, Environment and Life Sciences, Argonne National LaboratoryArgonne, IL, USA; ^3^Centre of Biological Engineering, University of Minho, Campus de GualtarBraga, Portugal; ^4^Mathematics and Computer Science Division, Argonne National LaboratoryArgonne, IL, USA; ^5^Computational Biology and Bioinformatics Group, Pacific Northwest National Laboratory (U.S. Dept. of Energy)Richland, WA, USA; ^6^Department of Computer Science, Ryerson Physical Laboratory, University of ChicagoChicago, IL, USA; ^7^Biology Department, Hope CollegeHolland, MI, USA; ^8^Computer Science Department, Hope CollegeHolland, MI, USA; ^9^Department of Mathematics, Statistics and Computer Science, Dordt CollegeSioux Center, IA, USA; ^10^Fellowship for Interpretation of GenomesBurr Ridge, IL, USA

**Keywords:** atomic regulon, clustering, gene expression analysis, transcriptomic data, *Escherichia coli*, hierarchical clustering, CLR, k-means clustering

## Abstract

Understanding gene function and regulation is essential for the interpretation, prediction, and ultimate design of cell responses to changes in the environment. An important step toward meeting the challenge of understanding gene function and regulation is the identification of sets of genes that are always co-expressed. These gene sets, *Atomic Regulons* (ARs), represent fundamental units of function within a cell and could be used to associate genes of unknown function with cellular processes and to enable rational genetic engineering of cellular systems. Here, we describe an approach for inferring ARs that leverages large-scale expression data sets, gene context, and functional relationships among genes. We computed ARs for *Escherichia coli* based on 907 gene expression experiments and compared our results with gene clusters produced by two prevalent data-driven methods: Hierarchical clustering and k-means clustering. We compared ARs and purely data-driven gene clusters to the curated set of regulatory interactions for *E. coli* found in RegulonDB, showing that ARs are more consistent with gold standard regulons than are data-driven gene clusters. We further examined the consistency of ARs and data-driven gene clusters in the context of gene interactions predicted by Context Likelihood of Relatedness (CLR) analysis, finding that the ARs show better agreement with CLR predicted interactions. We determined the impact of increasing amounts of expression data on AR construction and find that while more data improve ARs, it is not necessary to use the full set of gene expression experiments available for *E. coli* to produce high quality ARs. In order to explore the conservation of co-regulated gene sets across different organisms, we computed ARs for *Shewanella oneidensis, Pseudomonas aeruginosa, Thermus thermophilus*, and *Staphylococcus aureus*, each of which represents increasing degrees of phylogenetic distance from *E. coli*. Comparison of the organism-specific ARs showed that the consistency of AR gene membership correlates with phylogenetic distance, but there is clear variability in the regulatory networks of closely related organisms. As large scale expression data sets become increasingly common for model and non-model organisms, comparative analyses of atomic regulons will provide valuable insights into fundamental regulatory modules used across the bacterial domain.

## Introduction

The inference of gene function and regulation represent intertwined challenges in Systems Biology (Kitano, 2002). Regulon content often leads to the inference of functions for genes contained in the regulon, and assigned functions are often used to refine regulon structure. Microarray technologies (Young, 2000) accelerated the study of gene regulation by facilitating the production of thousands of expression datasets (Edgar et al., 2002), and next generation sequencing technologies massively increased the availability of reference genomes while also enabling the estimation of relative gene expression (Wang et al., 2009). Despite these advances, we still lack a complete understanding of gene function and regulation even in the most well studied bacterium, *Escherichia coli* (Kochanowski et al., 2013), which is the subject of thousands of phenotype experiments, gene expression datasets, and multiple regulation databases (Huerta et al., 1998; Salgado et al., 2013; Karp et al., 2014).

A key step in the inference of gene regulatory networks, and a valuable step in the functional annotation of genes, is the decomposition of a genome into sets of co-expressed genes. Today, three general methods exist for identifying sets of co-expressed genes: (i) clustering methods; (ii) transcription factor binding-site (TFBS) analysis (Rodionov, 2007); and (iii) *de novo* reverse engineering from expression data (De Smet and Marchal, 2010). Classic clustering methods, such as hierarchical clustering (Murtagh, 1985) and the centroid k-means clustering (Lloyd, 1982), aim to group sets of objects based on some criteria; when applied to the analysis of gene expression data, the aim is to group genes with similar expression profiles. TFBS tools, such as the popular RegPredict (Novichkov et al., 2010), infer regulons based on the presence of conserved upstream regions of DNA, which are presumed to be *cis*-regulatory elements. *De novo* reverse engineering methods use expression data to infer gene-to-gene regulatory interactions. One of the most commonly used methods, Context Likelihood of Relatedness (CLR), has been successfully applied to infer novel regulatory interactions (Faith et al., 2007).

Algorithms for computing co-expressed gene sets produce two different types of output: Regulons comprising a transcription factor and an associated set of regulated genes, or a set of co-expressed genes. The first type of output is produced by TFBS binding-site analysis and *de novo* reverse engineering methods to produce gene sets consistent with the classical definition of a regulon—genes are merged together into a set only if they respond to a common transcription factor. It is possible for a gene to appear in multiple sets if it responds to multiple transcription factors. This type of regulon information is valuable as a building block for assembling transcriptional regulatory networks and can be used, for instance, in deriving constraints to represent regulation in metabolic models (Shlomi et al., 2007; Chandrasekaran and Price, 2010). However, the overlap in their gene content and the resulting complexity in their interpretation make them less ideal for other applications of co-expressed gene sets.

Purely data-driven algorithms, such as hierarchical clustering or k-means clustering, can be used to produce the second type of output, sets of co-expressed genes that are not necessarily associated with a transcription factor. We propose to call the sets of co-expressed genes that are always ON or OFF together, *Atomic Regulons* (ARs). We define an AR as a set of genes that have essentially identical expression patterns, indicating a strong likelihood that they are functionally related (i.e., the genes are *always* expressed as a set). Each gene can be a member of only one AR; some ARs are represented by a single gene. Thus, a genome can be thought of as being comprised of ARs, with ARs considered to be the fundamental functional units of the cell. As the cell transitions from one functional state to another, it will activate some ARs, and deactivate others, with the functional states being defined by the set of active ARs. Cell states can be thought of as being organized hierarchically, with the ARs that represent core functions being constitutively expressed and the ARs that represent peripheral functions being expressed under specific conditions. In this way, analyzing expression patterns of ARs provide insights about gene functions and relationships among cellular systems.

The concept of atomic regulons has many useful applications. ARs are commonly used to provide insights into functions of orphan genes using the guilt-by-association principle, most prominently in resources such as STRING (von Mering et al., 2005). ARs are also used to plug gaps in metabolic reconstructions and models (Benedict et al., 2014). In addition, we recently applied ARs in the curation of regulatory network models to map regulons to stimuli (Faria et al., 2016). ARs make a statistical inference based on input data and are uniquely suited to these applications because: (i) they do not overlap in their gene content; (ii) all of their genes are co-expressed; (iii) they do not exclude genes that are co-expressed; and (iv) they may be computed reliably from tractable amounts of data.

Here we describe a new algorithm for computing ARs, which combines many of the advantages of the existing data-driven approaches, but integrates new evidence types including chromosome location and functional relationships to more quickly converge on a complete set of biologically meaningful ARs. We apply the new atomic regulon inference method to *E. coli*, which has large amounts of expression data that represent many environmental conditions. We compare the new *E. coli* ARs with those produced by existing data-driven approaches, curated sets of regulons mined from literature (RegulonDB), and with CLR-derived gene clusters. We then compute ARs for a set of four bacteria with increasing phylogenetic distance from *E. coli* to begin to understand the nature of conserved and unique ARs across organisms.

## Materials and methods

### Algorithm for computing atomic regulons

Our atomic regulon inference algorithm is unique from other approaches in that it begins by constructing draft atomic regulons (gene sets) using a combination of operon predictions and SEED subsystem technology (Overbeek et al., 2005). A subsystem is a set of related functional roles that represents the group of proteins involved in a biological process or pathway (e.g., protein biosynthesis or TCA cycle). In contrast to purely data-driven approaches, which start by forming gene clusters based only on available expression data, our approach applies expression data after initial gene clusters are formed in order to either extend or divide gene clusters to ensure they contain all co-expressed genes.

**Figure 1:**
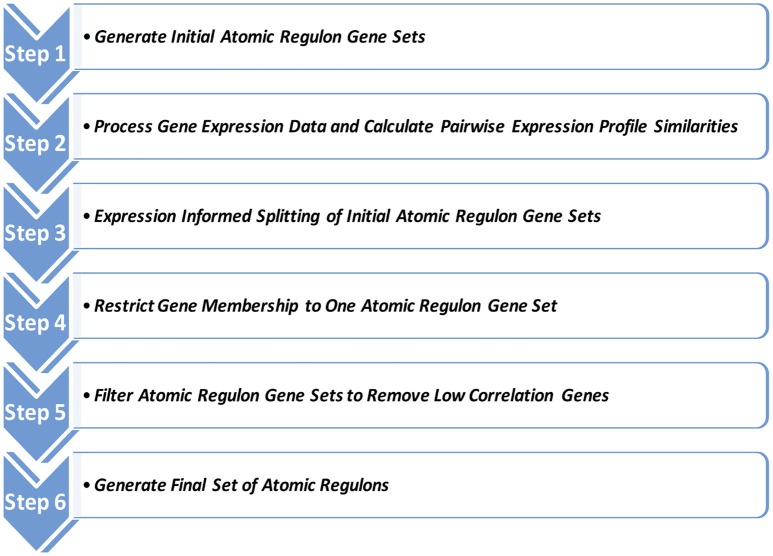
**Atomic Regulon Inference**. Six steps of Atomic Regulon (AR) inference algorithm. Step 1. Generate Initial Atomic Regulon Gene Sets. Initial clusters are proposed using gene clustering within putative operons and membership of genes within SEED subsystems. Step 2. Process Gene Expression Data and Calculate Pairwise Expression Profile Similarities. Integrate gene expression data, load the normalized data, and compute Pearson correlation coefficients. Step 3. Expression Informed Splitting of Initial Atomic Regulon Gene Sets. Split operon and subsystem-based clusters using the criterion that genes in a set must have pairwise expression data profiles greater than a set Pearson correlation coefficient (PCC) threshold. Step 4. Restrict Gene Membership to One Atomic Regulon Gene Set. Merge the clusters built from operons and subsystems then use the binary connections to form a single set of large clusters using transitive closure. This also ensures that no gene is a member of more than one cluster. Step 5. Filter Atomic Regulon Gene Sets to Remove Low Correlation Genes. Split the merged clusters based on a distance computed between every pair of genes. This corrects for genes with a low PCC value that may have been placed in a common cluster. Step 6. Generate Final Set of Atomic Regulons. Estimate the ON/OFF status of each cluster in any specific experimental sample by a simple voting algorithm using the ON/OFF estimates for the genes that make up the AR. This merged set becomes the final set of ARs.

Thus, the computation of ARs is derived from gene context information, functional annotation, and estimates of gene ON and OFF states from the expression data. The estimation of gene ON and OFF states is pre-computed as a separate step (see “Estimation of gene ON/OFF states” in Materials and Methods). Our AR inference process consists of six steps (Figure 1).

Step 1. Generate Initial Atomic Regulon Gene Sets
Compute a set of hypotheses in the form:
Genes G1 and G2 should be in the same atomic regulon
Initial clusters are proposed using two independent mechanisms: (i) gene clustering within putative operons; and (ii) membership of genes within SEED subsystems.
Step 2. Process Gene Expression Data and Calculate Pairwise Expression Profile Similarities
Integrate all available gene expression data for the genome, load the normalized data, and compute Pearson correlation coefficients (PCCs) for all possible gene pairs. PCCs are computed to provide a quantitative assessment of how similar the expression profiles are for each gene pair.
Step 3. Expression Informed Splitting of Initial Atomic Regulon Gene Sets
Split operon and subsystem-based clusters using the criterion that genes in a set must have pairwise expression data profiles greater than a PCC of 0.7. This ensures that the initial clusters contain genes that all share a substantial level of co-expression.
Step 4. Restrict Gene Membership to One Atomic Regulon Gene Set
Merge the clusters built from operons and subsystems as, at this point, genes may be members of more than one cluster. We convert each cluster into a set of binary connections between all genes in the cluster. We then use the binary connections to form a single set of large clusters using transitive closure (e.g., if A is connected to B and B is connected to C, then A is connected to C). This leads to a condition in which any two genes that are connected are in the same cluster. This also ensures that no gene is a member of more than one cluster.
Step 5. Filter Atomic Regulon Gene Sets to Remove Low Correlation Genes
Split the merged clusters based on a distance computed between every pair of genes using the formula
  $$\begin{array}{l} Distance\;=\;\frac{\left(2-\left(PCC+1\right)\right)}{2} \end{array}$$
This corrects for genes with a low PCC value that may have been placed in a common cluster. New sub-clusters are formed by taking the two closest genes (based on the above defined distance) within the initial merged cluster and adding other genes to the growing sub-cluster. At each point, the gene with the minimum average distance to genes in the growing sub-cluster is added to the sub-cluster, until no such gene exists with an average distance less than or equal to 0.25. If this simple accretion algorithm produces a single sub-cluster, no splitting is required. If not, the sub-clusters become the close to final AR gene sets.
Step 6. Generate Final Set of Atomic Regulons
Estimate the ON/OFF status of each cluster in any specific experimental sample by a simple voting algorithm using the ON/OFF estimates for the genes that make up the AR (see Estimation of gene ON/OFF states). We then merge these AR gene sets if they have identical ON/OFF expression profiles across all experimental conditions. It is important to note that the resulting set of atomic regulons is not comprehensive (i.e., not all genes are placed into an AR), but this set attempts to capture many of the operational groups of genes. This merged set becomes the final set of ARs.

The source code for the AR inference algorithm is available on GitHub (https://github.com/ModelSEED/atomic_regulons). Additionally, a service for AR inference has been implemented as a tool named “Compute Atomic Regulons” in the DOE Systems Biology Knowledgebase (KBase). Compute Atomic Regulons can be accessed at https://narrative.kbase.us. This service allows users to upload expression datasets and compute ARs for their genomes of interest. A tutorial detailing how to compute ARs in KBase is available in supplementary material.

### Estimation of gene ON/OFF states

Our AR inference algorithm requires us to compute the correlation of expression for all genes across all available expression datasets. In this computation, we use expression values to assign all genes in all conditions to one of three possible states: ON, OFF, and UNKNOWN. These gene states are calculated for all genes in two steps.

Step 1. Initial Estimates of ON/OFF Calls Using an A Priori Identified Set of “Always ON” Genes
  - Determine the threshold for a gene to be considered ON based on the normalized expression of genes annotated with functions that are expected to be universally active. In total, we identified 80 functional roles from the SEED as universally active, largely from translation and transcription (see Supplementary Table S1). We then consider the genes that implement these roles as ON.
  - Empirically set initial ON/OFF calling thresholds for each experiment. The “ON” threshold for experiment *i*, called *N*_*i*_, is set as the 10th percentile of observed gene expression values for “always ON” genes in experiment *i*. The “OFF” threshold for experiment *i*, called *F*_*i*_, is set as the 80th percentile of observed gene expression values that are below *N*_*i*_ in experiment *i*.
  - Update initial ON/OFF calling thresholds for each experiment by computing the difference, *D*_*i*_ = *N*_*i*_–*F*_*i*_, in thresholds for each experiment, then finding the 25th percentile of *D*_*i*_ across all experiments, *D*_25*th*_. For any experiment *i* where *D*_*i*_ < *D*_25*th*_, set *F*_*i*_ = *N*_*i*_–*D*_25*th*_. This ensures that the “ON” and “OFF” calling thresholds are never too close together for a particular experiment.
  - Using the updated ON/OFF calling thresholds for each experiment (*N*_*i*_ and *F*_*i*_), make initial ON/OFF calls for each gene in experiment *i* by classifying any gene expression value less than *F*_*i*_ as OFF, greater than *N*_*i*_ as ON, and between *F*_*i*_ and *N*_*i*_ as UNKNOWN.
Step 1. Updating Gene-Specific ON/OFF Calls Using Gene Sets to Ensure Maximal Consistency
  - Construct draft sets of genes that are expected to be co-expressed with a high degree of confidence. Sets are constructed from: (i) operons; and (ii) subsystems.
  - Vote within each gene set to determine the ON/OFF status of the entire set in each experiment based on majority rule. For example, if a set of four genes has two genes initially called ON, one UNKNOWN, and one initially called OFF, we update the calls for all genes in the set to ON since that is the majority of the initial calls. Ties (e.g., two ON and two OFF or all UNKNOWN) are classified as UNKNOWN.

### Gene expression data

Gene expression data were collected from the Gene Expression Omnibus (GEO) (Edgar et al., 2002) and M3D databases (Faith et al., 2008), as well as from the laboratory of Dr. Paul Dunman in the case of *S. aureus*. Expression data were downloaded in the form of Affymetrix GeneChip® cell intensity (CEL) files. For each organism, the expression data from the CEL files were background corrected, normalized and summarized using Robust Multichip Averaging (Irizarry et al., 2003) as implemented in R/Bioconductor (http://www.bioconductor.org/) using the *rma* function default settings. In addition, the probe sets for each Affymetrix GeneChip were mapped to gene identifiers in the SEED genome database (Tintle et al., 2012; Overbeek et al., 2014).

### Computation of hierarchical and k-means clusters

In order to compare the performance of ARs with k-means and hierarchical clustering analyses, we applied these methods to the normalized gene expression values across all experiments to generate 646 clusters, the same number of clusters identified using our AR approach for the purpose of illustration. K-means clusters were calculated with k set to 646 clusters using the Lloyd-Forgy algorithm (Lloyd, 1982) in R (Team, 2015). This approach is a non-standard use of the k-means clustering algorithm; the value of k was set to match the number of ARs for the sake of comparison. This clustering approach defines clusters by minimizing the Euclidian distance between individual points and cluster centers and is sensitive to variations in true cluster size and variance distributions. For hierarchical clustering analysis, a Euclidean distance matrix was calculated between genes based on their normalized expression values across all experiments. We performed hierarchical clustering on this distance matrix using the average algorithm in R (Murtagh, 1985), which is equivalent to the unweighted pair group method using arithmetic mean (UPGMA). This clustering approach begins with each gene assigned to its own cluster and proceeds through an iterative process of joining the two nearest clusters together until all genes are linked into a hierarchical tree. We cut this tree at a given height in order to yield 646 clusters.

### Assessing similarity of gene sets

We performed comparisons between gene sets produced by our AR inference algorithm and standard data-driven clustering algorithms to regulons in RegulonDB. We use the Jaccard coefficient, which measures similarity between finite sample sets. This coefficient is defined as the size of the intersection divided by the size of the union of the sample sets

$$\begin{array}{l} Jaccard\;coefficient\;\left(RegDB,Rx\right)\;=\;\frac{\left|RegDB\;\cap \;Rx\right|}{\left|RegDB\;\cup \;Rx\right|} \end{array}$$

where *RegDB* is a set of genes comprising a single regulon from the RegulonDB database, and *Rx* is a gene cluster inferred by one of the clustering algorithms being evaluated.

Additionally, we performed comparisons of ARs for *E. coli* with ARs for four different organisms, limiting our comparison to functional roles contained in SEED subsystems that occurred both in *E. coli* and the other organisms. For each atomic regulon in *E. coli* (*Ae*), we computed the set of relevant functional roles occurring in *Ae*, calling this set *Re*. Then, for each atomic regulon occurring in one of the other genomes (*Ax*), we considered the set of relevant roles occurring in *Ax*, calling this set *Rx*. We then computed the Jaccard coefficient for R*e* and R*x*.

### Context likelihood of relatedness (CLR) algorithm

We performed a check on the gene contents of each regulon using the *Context Likelihood of Relatedness (CLR)* algorithm developed by the Gardner group at Boston University. Given a set of gene expression data, CLR predicts transcriptional regulatory relationships (Faith et al., 2007). The CLR algorithm belongs to a category of regulatory network inference algorithms that uses mutual information (MI) to analyze correlations in gene expression. In brief, the higher the MI score between two genes, the greater the information we derive on the expression states of the first gene from the pattern of states in the other, and therefore the greater the likelihood that one of the genes is directly or indirectly regulating the other, or that both genes are being regulated together. Our CLR calculations were done on the DeGNServer website, where the CLR algorithm has been parallelized to handle large gene sets (Li et al., 2013). A CLR score was recorded for each possible unique gene pair in *E. coli* (each possible gene-to-gene connection), resulting in a total of 9,367,956 CLR scores.

We discarded all but the top 0.765% of the CLR scores, representing all scores with values that were at least four standard deviations above the mean score. All gene pairs associated with these top CLR scores are then said to have support from CLR. We used these regulatory interactions inferred by CLR to validate and assess the ARs inferred by our algorithm and gene sets produced by the clustering algorithms at the same level of granularity. In this validation, we calculated the fraction of all possible gene pairs in each AR that have a regulatory interaction also predicted by CLR. For example, if an AR includes three genes, then it has three possible gene pairs (i.e., AB, AC, CB). If CLR predicted a regulatory interaction between two of these pairs (i.e., AB, AC), then the fraction of supported interactions would be 0.667 (67% support). See Supplementary Information for a full treatment of the method.

### Prediction of operons from genome sequence and gene calls

We predict operons from genomic data to serve as initial gene sets for our AR inference algorithm. In our operon prediction approach, sets of genes in the same strand within 200 base pairs up and down stream of each other were placed into the same operon. This mirrors the existing standard algorithms for operon prediction (Salgado et al., 2000).

## Results

### Characteristics of *E. coli* K-12 atomic regulons

Atomic regulons were computed for *E. coli* using the AR inference algorithm and expression data from the M3D dataset. *E. coli* was selected for initial construction of atomic regulons because it has the largest compendium of consistent gene expression data that is currently available, comprising microarray data from 907 experiments. The computation was performed in approximately 12 min on a single dual-core CPU. Four metrics were used to characterize the set of ARs produced for a genome: (i) the total number of genes assigned to ARs; (ii) the number of ARs computed; (iii) the number of genes that were found to be always ON; and (iv) the number of genes found to be always OFF across all experiments.

A summary of the results of the inference pipeline for *E. coli* is shown in **Table 2**, and the full set of atomic regulons is available in Supplementary Table S2. For *E. coli*, 2604 genes are assigned to 646 multi-gene ARs, corresponding to approximately 60% of its genome. The largest ARs in the set contain 292 and 69 genes, representing the set of genes that are always expressed or are never expressed in the experimental conditions represented by the expression array data, respectively. The largest atomic regulon with variable expression in our available data is comprised of 52 genes, which are primarily related to the functions of motility and chemotaxis. The remaining 644 ARs contain less than 52 genes each, with an average size of 3.48. Approximately 85% of multi-gene ARs contain 5 or less genes, with 328 ARs containing only 2 genes. Again, we deployed our AR computation algorithm into KBase, and we demonstrate this method at work in computing ARs for *E. coli* in a KBase Narrative (https://narrative.kbase.us/narrative/ws.14533.obj.1).

### Comparison to clusters produced by hierarchical clustering and k-means clustering

**Figure 2:**
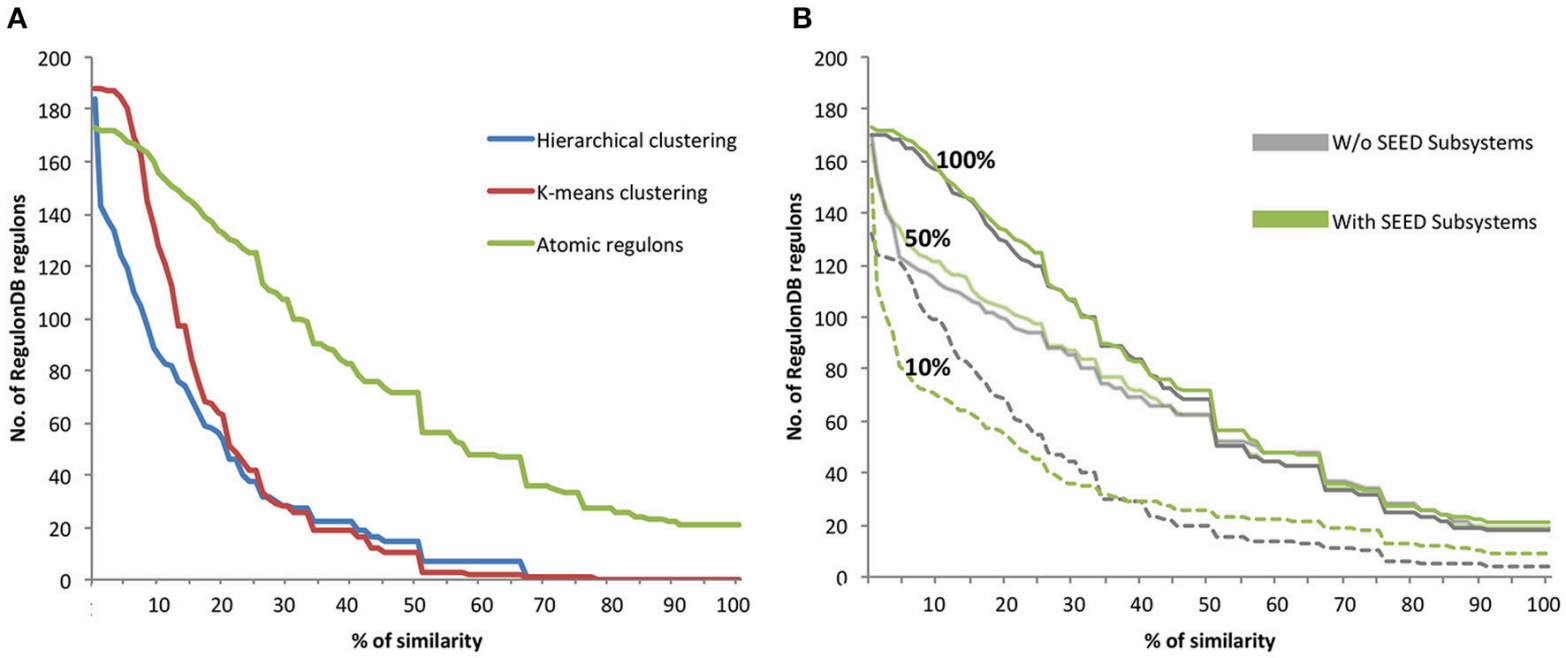
**Comparison of RegulonDB regulons with hierarchical clustering, k-means clustering and atomic regulons. (A)** The Jaccard coefficient comparing *E. coli* RegulonDB regulons vs. the clustering methods is shown as a percentage of similarity. **(B)** Comparison AR similarity to RegulonDB regulons with and without inclusion of SEED subsystems using 100, 50, and 10% of experiment data.

The AR inference algorithm was compared to data-driven clustering algorithms by assessing the consistency of gene sets to both a curated set of regulons present in the database RegulonDB and to interactions predicted by the CLR algorithm. Each of the gene sets was compared to the reference set of regulons for *E. coli* found in RegulonDB through the calculation of a Jaccard coefficient (details in the Materials and Methods). The analysis revealed that the co-expressed gene sets generated by our AR inference algorithm had a higher level of similarity to the gold standard regulons in RegulonDB than the gene sets generated by either hierarchical or k-means clustering (Figure 2A). The data driven clustering methods produce gene sets that are much less consistent; less than 8% of gene sets show 50% or more similarity to RegulonDB regulons. In contrast, approximately 50% of the RegulonDB regulons show a >50% similarity with the gene sets generated by our AR inference algorithm, and approximately 20% have a similarity >70%.

Additionally, we performed a similar analysis to investigate the impact of the inclusion of SEED subsystems in the AR inference algorithm. We compared AR similarity to RegulonDB by computing ARs with and without SEED subsystems using three different subsets of the available experimental data: (i) Full dataset of 907 experiments; (ii) 403 randomly selected experiments (50% of the data); and (iii) 91 randomly selected experiments (10% of the data) (Figure 2B). With the entire dataset of E. coli experiments, we observe only a small improvement in the similarity with RegulonDB with the inclusion of SEED subsystems. For the subset comprising of 50% of the data, we observe a slightly larger improvement with the inclusion of SEED subsystems when compared to the full dataset. These results are corroborated by the analysis conducted on **Figure 4**, in which we conclude that starting at 60% of the data, the improvements in our AR computation grow markedly slower.

For the subset comprising only 10% of the *E. coli* data, we see the largest difference in similarity with RegulonDB. In this case, the results are mixed. When the regulons have a larger degree of similarity with RegulonDB, they tend to be more accurate when generated with subsystems. When regulons have a lower degree of similarity with RegulonDB, they tend to be less accuracy when generated with subsystems. This result is due to the fact that the use of SEED subsystems will generally increase the average size of the regulons, as well as the fraction of the genome that is included in ARs. Note for example, that we have 150 ARs with at least some overlap with RegulonDB when subsystems are used, vs. only 130 without. Thus, part of the observed decline in similarity at the lower end of the comparison curve is due to having more (and larger) regulons used in the comparison. This also emphasizes the vital role that the expression data plays in the AR algorithm. When limited data is available, it could make sense to apply our algorithm with and without subsystems and compare the results.

CLR is a mutual-information-based approach for the inference of gene regulatory networks from gene expression data (Faith et al., 2007). The CLR algorithm has been applied extensively to validate *E. coli* regulatory interactions and to identify missing links in the *E. coli* regulatory network. Also, CLR has performed well in DREAM challenges (dreamchallenges.org/publications/) when compared against other inference algorithms. We applied CLR to the same set of expression data that was used to build the ARs and clustering algorithm gene sets for *E. coli*, computing CLR scores for every possible pair of genes in *E. coli*. These scores quantify the level of mutual information found between the expression profiles of each pair of genes, with higher scores meaning more mutual information and thus a greater chance that one gene in the pair is co-regulated with or regulates the other gene in the pair.

**Figure 3:**
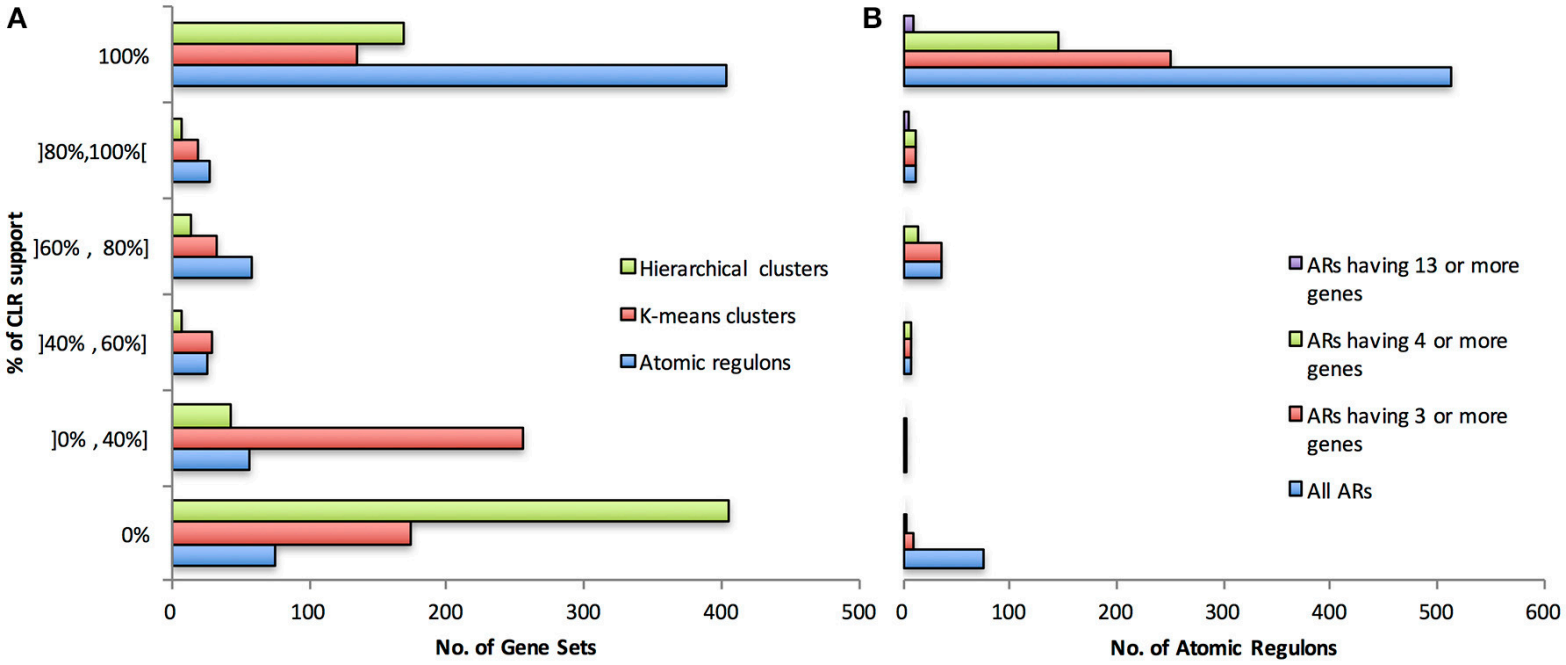
**Degree of CLR support. (A)** CLR support compared between our ARs, and ARs produced via k-means clusters and hierarchical clusters. **(B)** CLR support for our AR construction method, broken down by different AR sizes.

The ARs generated by our algorithm and gene sets generated from hierarchical clustering and k-means clustering were assessed by binning ARs and gene sets into different ranges of support from CLR (Figure 3A). The highest category of support for an AR or gene set is one in which all of the gene pairs in the set have a high CLR mutual information score with at least one other member of the gene set; 62% of ARs fall into this category, whereas 26 and 21% of hierarchical and k-means clustering gene sets fall into this category, respectively. We evaluated if a bias existed for the agreement between CLR and our AR inference algorithm based on AR size (Figure 3B). Given that larger ARs involve more possible gene pairs and thus more possible interactions that need to be validated by CLR, it was possible that there would be lower CLR support for the larger ARs. However, all 15 of the ARs with 13 or greater gene members were found in the two highest categories of CLR support (80–100 and 100%). More details on this analysis are available in the supplementary material. In particular, see Supplementary Tables CS-1, CS-2A–C.

### Sensitivity of AR inference algorithms to the amount of available expression data

While advances in sequencing technology have led to the ability to produce expression data for non-model organisms, it remains the case that *E. coli* has the largest amount of expression data available among bacteria to date. The expression data have also been obtained in a large number of experimental conditions. This sets up a seemingly ideal case for the calculation of atomic regulons. However, most organisms will not have as much data available as *E. coli*, and it may be possible to obtain meaningful ARs with less expression data. Thus, we studied the impact of decreasing amounts of expression data on the inference of atomic regulons. We randomly selected different subsets of the *E. coli* data, repeated the AR analysis with each data subset, and compared the end results. For our subsets, we selected all increments of 10% of the available data (i.e., considering 10, 20, 30%, etc.). We repeated the analysis 100 times for each data size, selecting a different random subset of data with each simulation.

We evaluated the atomic regulons computed in each simulation based on four metrics. The first metric was the *number of genes* assigned to ARs of size two or greater. If insufficient data are available, natural noise in gene expression patterns will overwhelm any correlations that exist between genes, preventing the consolidation of ARs based on correlation of gene expression. Thus, the expectation is that fewer genes will be integrated together into multi-gene ARs. The second metric was the *number of multi-gene ARs* computed. As before, the signal to noise ratio that occurs with smaller amounts of data will prevent some multi-gene ARs from forming. Additionally, with fewer experiments, fewer genes will be differentially expressed. This will prevent some large ARs from being broken up into smaller ARs. The third and fourth metrics are the *number of genes that are always ON* and the *number of genes that are always OFF*, respectively. Fewer experiments may represent fewer experimental conditions and fewer differentially expressed genes, leading to an expectation of more genes that are either always ON or always OFF.

**Figure 4:**
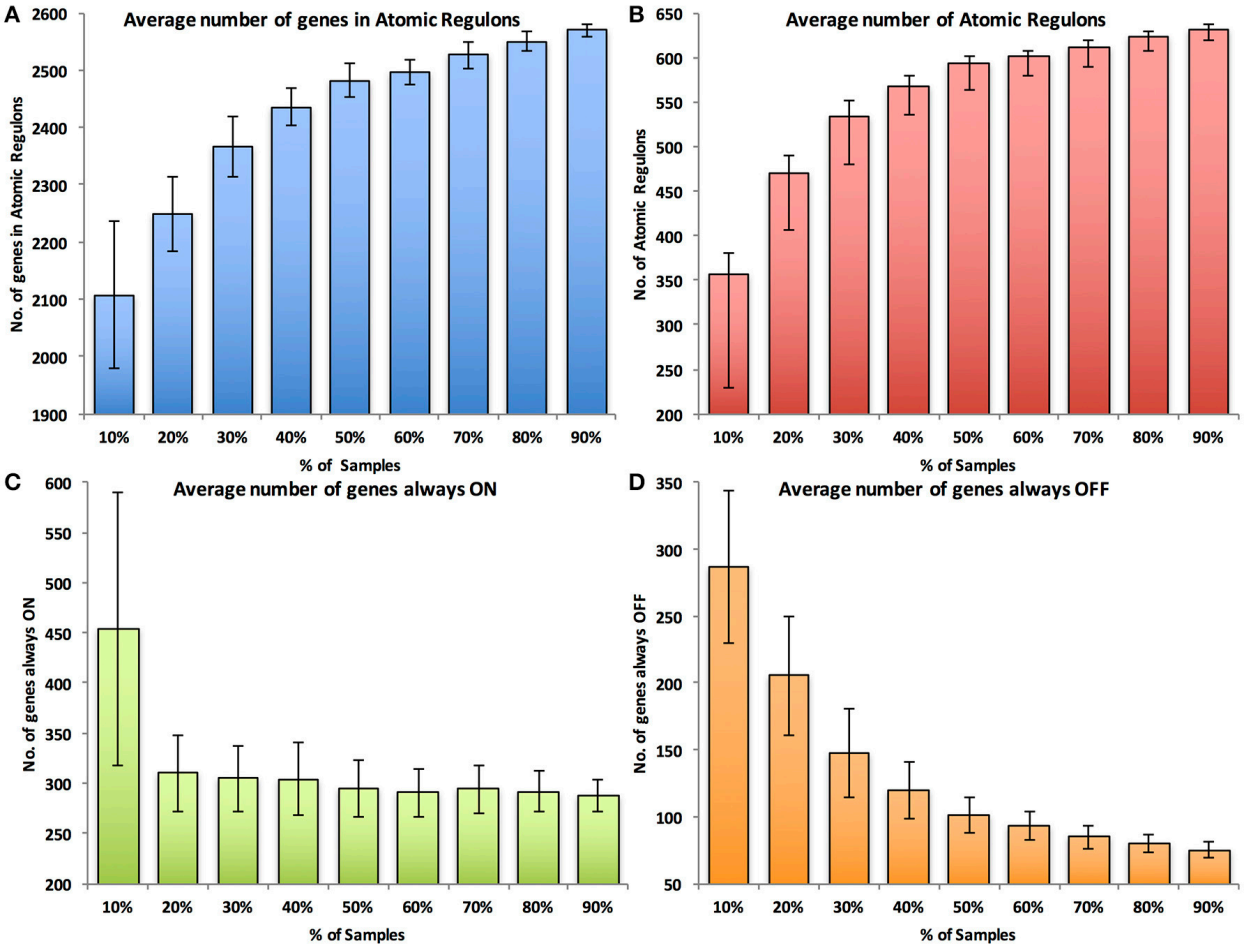
**Sensitivity analysis of Atomic Regulon inference for ***Escherichia coli*** K-12. (A)** Average number of genes in atomic regulons. **(B)** Average number of atomic regulons. **(C)** Average number of genes always ON **(D)** Average number of genes always OFF. Standard deviation error bars represent the variation across 100 data set randomizations from random sampling of experiments.

The results of the random sensitivity analysis support the expectations. As the amount of available data increases, the number of genes in ARs (Figure 4A) and the total number of ARs increase (Figure 4B). Additionally, the numbers of always ON genes (Figure 4C) and always OFF genes (Figure 4D) decrease with increasing amounts of expression data. Interestingly, in all cases, large improvements in each of the metrics are observed as the amount of data used increases from 10 to ~60% of the available data. Continued improvements in all metrics are observed until 100% of the data is used, but the improvements grow markedly smaller as more than 60% of the data is considered. We also performed a simple 2-fold cross validation of the data, randomly splitting the 907 experiments for *E. coli* into equal non-overlapping sets (Table 1). When we compare the regulons computed from these two data subsets, the average Jaccard coefficients (0.83 ± 0.31 and 0.80 ± 0.35, mean and standard deviation) were nearly identical to the comparison of atomic regulons computed from the full dataset to either of the subsets (0.81 ± 0.35 and 0.80 ± 0.37 respectively). This result shows that the atomic regulons are very similar when only half the available experimental data is used.

**Table 1:** **Average Jaccard similarity coefficient between each set of atomic regulons from 2-fold the cross validation**.

	**Set1**	**Set2**	**All ARs**
Set1	1	0.80 ± 0.35^*^	0.89 ± 0.26
Set2	0.83 ± 0.31	1	0.92 ± 0.19
All ARs	0.81 ± 0.35	0.80 ± 0.37	1

* *Mean ± Standard Deviation*.

These results show that there is enough existing gene expression data for *E. coli* to enable the generation of high quality ARs, capturing a large portion of the atomic regulon space for this organism. Further, it is possible to compute ARs for *E. coli* with approximately the same quality using a much smaller amount of data.

### Computation of atomic regulons across taxa

Studies of the evolution of bacterial transcriptional regulatory networks show conservation of regulatory modules/motifs and that gene co-regulation tends to be more conserved than regulatory genes and mechanisms. This conservation is observed across large phylogenetic distances for organisms with similar lifestyles (Madan Babu et al., 2006). Atomic regulons should be well suited to explore these trends. Thus, the AR inference algorithm was applied to study the consistency of gene co-regulation across five diverse genomes. ARs were computed for four additional organisms that have sufficient high-quality expression data available: *Shewanella oneidensis* MR-1, *Pseudomonas aeruginosa* PAO1, *Thermus thermophilus* HB8 *and Staphylococcus aureus* subsp. aureus Mu50 (organisms are ordered approximately from the closest to the farthest in terms of phylogenetic distance). To minimize noise, all the experimental datasets were selected by considering the same microarray platform with the same process of applied data normalization (see Materials and Methods). However, it should be noted that any source of expression data that is converted into normalized ON/OFF estimates can be used to infer ARs, ensuring that the technique is applicable to the increasing number of RNA-Seq experiments available for model and non-model organisms and to considering emerging data in combination with the rich amount of existing microarray expression data. The basic metrics describing ARs for each of the organisms are shown in Table 2. We demonstrate the application of our method to computing ARs for these genomes in a KBase Narrative (https://narrative.kbase.us/narrative/ws.14533.obj.1).

**Table 2:** **Atomic regulon statistics for ***Shewanella oneidensis*** MR-1, ***Pseudomonas aeruginosa*** PAO1, ***Thermus thermophilus*** HB8, and ***Staphylococcus aureus*** subsp. aureus Mu50 and ***Escherichia coli*** K-12**.

	**Array datasets**	**No. ARs**	**Genes in ARs**	**Genes always ON**	**Genes always OFF**	**Genome size**
*Escherichia coli* K-12	907	646	2604 (60%)	292 (6.8%)	69 (1.6%)	4309
*Shewanella oneidensis* MR-1	245	335	1559(37%)	265 (6.4%)	32 (0.8%)	4167
*Pseudomonas aeruginosa* PAO1	236	423	2427(43%)	557 (9.8%)	78 (1.4%)	5682
*Thermus thermophilus* HB8	543	196	1422(63%)	692 (30.9%)	27 (1.2%)	2239
*Staphylococcus aureus* subsp. aureus Mu50	852	397	1749(63%)	447 (16.1%)	28 (1%)	2770

The descriptive statistics for the ARs from the five organisms suggest interesting trends. It appears that there is a correlation between the number of expression experiments available for an organism and the percentage of genes included in a multi-gene AR. For both *T. thermophilus* and *S. aureus*, the same proportion of genes were included as for *E. coli*. Each of these have a number of experiments corresponding to greater than 60% of the data available for *E. coli*, consistent with the analysis above where subsets of the data were used to calculate ARs for *E. coli*. The organisms with fewer expression experiments (~26% of the experiments available for *E. coli*) have a lower percentage of genes included in ARs. Given the caveat that the number of experiments does not necessarily reflect the number of unique environmental conditions represented in the set of experiments, it is interesting to speculate that more experimentation in these organisms would lead to inclusion of more genes in the calculated ARs. However, it is also apparent that the number of genes considered to be ON or OFF varies greatly, and that two of the three genomes with the highest number of ON genes have the highest number of experiments associated with them. This could reflect the diversity of the conditions represented by the experiments that leads to genes failing to be differentially expressed, the quality of the available expression data, or limitations of establishing thresholds for the ON/OFF state of genes in those organisms.

**Figure 5:**
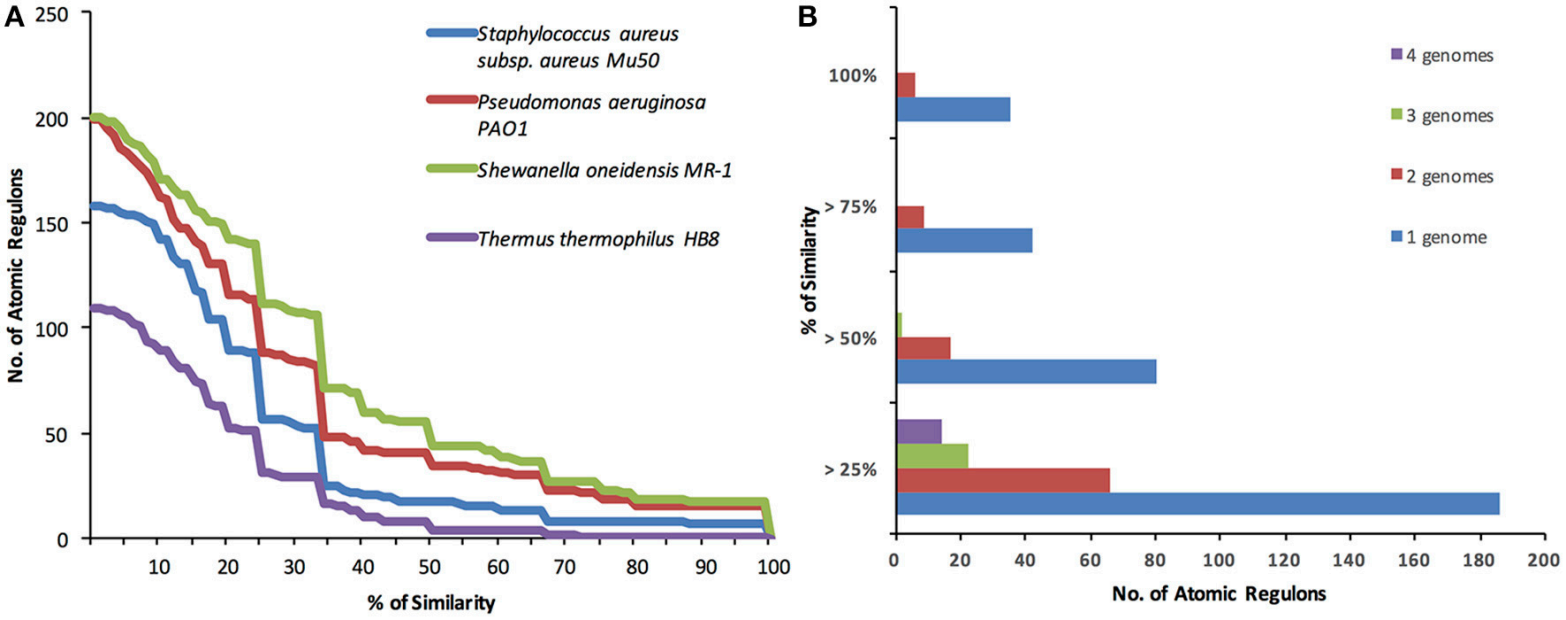
**Comparison of ***E. coli*** Atomic Regulons vs. all other ARs for ***Shewanella oneidensis*** MR-1, ***Pseudomonas aeruginosa*** PAO1, ***Thermus thermophilus*** HB8 ***and Staphylococcus aureus*** subsp. aureus Mu50. (A)** The % of similarity is given by the Jaccard coefficient, which is defined as the size of the intersection divided by the size of the union of the sample sets. **(B)** The % of similarity is given by the Jaccard coefficient, which is defined as the size of the intersection divided by the size of the union of the sample sets. Jaccard coefficients computed for each *E. coli* AR across all combinations of four, three, and two genomes.

In order to compare the ARs inferred for the different organisms, Jaccard coefficients were computed for each AR in *E. coli* vs. all ARs in each of the four other genomes. The distribution of these computed coefficients for each genome analyzed reveals that regulation appears to be more similar in genomes that are closer to *E. coli* both phylogenetically and in terms of lifestyle (Figure 5A). The only Gram positive genome we included in our study, *S. aureus*, was, as expected, a distant genome in terms of AR variation. The most distant genome in terms of AR variation was *T. thermophilus*, which, despite being “Gram negative,” is phylogenetically distant and found in environments that are highly distinct from that of *E. coli*. Another interesting observation is how much variation exists in AR content between *E. coli* and the closest genomes analyzed, *S. oneidensis* and *P. aeruginosa*. Although these genomes are close to *E. coli* phylogenetically, only a small fraction of their atomic regulons have high compositional similarity. In contrast to expectations, the composition of co-regulated gene sets appears to be highly variable among closely related organisms. This result could support the notion that regulation is a highly adaptable system in the cell, but experimental studies specifically dedicated to this type of comparative analysis are needed in order to confirm this result.

### Conservation of atomic regulons across taxa

In addition to conducting pairwise comparisons between *E. coli* and four other genomes, we focused on the specific atomic regulons that display the greatest consistency across the greatest number of genomes. To do this, we compared the Jaccard coefficients computed for each *E. coli* AR across all combinations of four, three, and two genomes. For each regulon, we retain the lowest Jaccard coefficients among the genomes being compared, with that coefficient being indicative of the compositional similarity of the AR among the full set of genomes (Figure 5B).

The analysis revealed 35 ARs with 100% compositional similarity between *E. coli* and at least one other genome in our set (the distribution of these 35 ARs across all genomes compared can be estimated from Figure 5A). This number is larger than the highest number of 100% identical ARs from our pair-wise AR comparisons (18), which indicates that the ARs with 100% similarity between *E. coli* and each individual genome have significant differences (i.e., different ARs were identical in each species). No identical regulons across *E. coli* and the four other genomes were observed, consistent with the large number of differences found between the ARs of *E. coli* and the ARs for *T. thermophilus* and *S. aureus*.

There were nine atomic regulons with at least 75% similarity across *E. coli* and two other genomes (Table 3). All ARs in Table 3 were conserved with and were most similar to ARs in *S. oneidensis*, the organism with the smallest phylogenetic distance to *E. coli* in this study. The two largest ARs conserved among *E. coli* and two other genomes are both functionally related to cellular respiration. The largest was compositionally identical across three genomes (*E. coli, S. oneidensis*, and *P. aeruginosa*) comprising 13 genes, most of which are annotated with functions related to the NADH-ubiquinone oxidoreductase chain. All genes in this AR are members of the SEED Subsystem “Respiratory Complex I.” These functions have been characterized in *E. coli* and are responsible for proton translocation in the electron transport chain (Weidner et al., 1993). The gene order is also highly conserved when compared to other bacterial genomes. Studies report these functional roles to be present in *T. thermophilus* (Yano et al., 1997), however, these functions were grouped into a much larger AR in this study, possibly due to the lack of expression data needed to decompose this large AR. The second largest identical AR is comprised of seven functional roles involved in the biogenesis of c-type cytochromes. These functional roles have been described in *E. coli* (Thöny-Meyer et al., 1995) and each were present in the *S. oneidensis* and *P. aeruginosa* atomic regulons. None of the respiratory chain associated functional roles in the two largest ARs identified through this analysis were present in *S. aureus*, consistent with known diversity of respiratory chain components among bacteria.

**Table 3:** **Atomic regulons similarity >75% across ***E.coli*** and two other genomes**.

**AR ID**	**AR size**	**Associated genomes**	**Functional role summary**
15	13	*S. oneidensis* MR-1	NADH-ubiquinone oxidoreductase chain
		*P. aeruginosa*	
		PAO1	
54	7	*S. oneidensis* MR-1	Biogenesis of c-type cytochromes
		*P. aeruginosa*	
		PAO1	
57	6	*S. oneidensis* MR-1	Tryptophan synthesis
		*S. aureus*	
112	5	*S. oneidensis* MR-1	Phosphate transport system
		*S. aureus*	
316	3	*S. oneidensis* MR-1	Molybdenum transport system
		*P. aeruginosa*	
		PAO1	
362	2	*S. oneidensis* MR-1	Heat shock proteins
		*S. aureus*	
398	2	*S. oneidensis* MR-1	Paraquat-inducible proteins
		*P. aeruginosa*	
		PAO1	
500	2	*S. oneidensis* MR-1	Ribonucleotide reductase
		*S. aureus*	

The third and fourth largest ARs shared among *E. coli* and two other genomes are both shared with *S. oneidensis* and *S. aureus*. One AR comprising six functional roles is associated with tryptophan synthesis. In *T. thermophilus* and *P. aeruginosa*, only 40% of the functional roles are present in the corresponding AR. These results are consistent with research on the evolution and dynamics of the tryptophan biosynthesis pathway that found some functional roles are conserved across multiple organisms, while others were lost due to events such as operon splitting or gene fusions (Xie et al., 2003). The fourth largest AR contains functional roles associated with phosphate transport in *E. coli* (Amemura et al., 1985), and it was 100% identical to the AR identified in *S. oneidensis* and 80% in *S. aureus*. All results from the comparative analysis of ARs are reported in Supplementary Table S3.

To aid in genome annotation efforts, we integrated the computed ARs for this study into the SEED database. They can be accessed in the interface showing information for all genes that are members of a computed AR: http://pubseed.theseed.org/?page=AtomicRegulon&genome=all. Additionally, we implemented the AR inference algorithm in KBase (http://www.kbase.us), allowing the computation of AR for any genome of interest given an expression data set (see Material and Methods).

## Discussion

Atomic regulons represent fundamental regulatory units of a cell, namely, the sets of genes that are always co-regulated. We have demonstrated a new method for the inference of atomic regulons that outperforms purely data-driven clustering methods for deriving sets of co-regulated genes. The approach relies on the formation of putative atomic regulons based upon operonal organization of genes and the highly curated sets of associated functions for genes (subsystems) used in the SEED and PATRIC databases (Overbeek et al., 2014; Wattam et al., 2014). These serve as high quality starting points that allow for the efficient determination of biologically meaningful co-regulated sets of genes. The inference algorithm is impacted by the current estimate of SEED Subsystems that are continuously improved by the SEED annotation team. We apply SEED subsystems to build and refine our initial gene clusters, meaning that errors in the assignment of genes to subsystems can potentially lead to errors in initial clusters (which is mitigated by the concurrent use of expression data). More critically, clusters involving primarily genes that are not assigned to SEED subsystems will not benefit from this aspect of the algorithm. Additionally, our algorithm for setting the “ON” threshold for gene activity depends on the identification of a set of genes in the genome that are expected to be universally active. We currently identify these genes based on the functional roles assigned to them by the SEED. If these genes are not accurately annotated by the SEED, it will impact our ability to set an accurate “ON” threshold for gene activity.

Assessments of the gene sets produced from the AR inference algorithm and two popular clustering algorithms suggest that using a biologically meaningful starting point rather than clustering based solely on expression data produce co-regulated gene sets that are much more consistent with independent measures of gene regulatory interactions. This includes the calculation of the Jaccard coefficient for each AR and that of an independent information-theoretic method, CLR; atomic regulons were shown to be more consistent with gold-standard regulons found in RegulonDB and with mutual information coefficients between pairs of genes as determined by CLR. Further, the AR inference algorithm is robust to varying amounts of expression data and can be applied to diverse organisms. These properties make the described AR inference algorithm attractive for addressing questions of the regulation of fundamental cellular processes, the interactions amongst those fundamental processes, and the conservation of the processes across cellular life.

The atomic regulons inferred for *E. coli* constitute a large portion of the genes in the organism, with most ARs comprising 3 genes, on average. The two largest ARs also represent the two gene sets whose genes are always ON and always OFF in the set of expression experiments studied; we refer to these as *static ARs*. The 292 genes comprising the static AR that is universally expressed are primarily annotated with functions related to core cellular machinery. A large fraction of the genes code for ribosomal proteins and tRNA synthetases. These functions are considered to be constitutive, and were selected to set the threshold for active gene expression used in our ON/OFF calling algorithm. The remaining genes were classified as constitutively expressed and merged into the static AR as part of the inference algorithm. These functions are associated with core cellular machinery, such as DNA synthesis and ATP synthetases. Additionally, multiple genes with unknown functions are members of this atomic regulon. The 69 genes comprising the static AR that are not expressed are poorly annotated, with 63% annotated as hypothetical, putative or uncharacterized. These genes are inactive in all conditions studied, meaning they likely contribute little to cell fitness in any of the conditions tested. It is expected that the static ARs would break apart as more conditions are represented in the expression experiments. Our sensitivity analysis revealed that the ARs were somewhat insensitive to random reductions in the *E. coli* dataset, until those reductions exceed 50% of the dataset. This said, it is the diversity of experimental conditions included in the dataset that has the most significant impact on the accuracy of predicted regulons (Nicolas et al., 2012), and a simple count of the number of datasets is not necessarily an effective measure for this diversity. Unfortunately, we lack sufficient metadata for available expression datasets to rigorously explore the relative impact of experimental diversity on regulon quality.

An initial assessment of the conservation of atomic regulons across a diverse set of bacterial organisms reveals several interesting observations. First, it is clear that there is great variability in the membership of atomic regulons for different organisms. None of the ARs were fully conserved among *E. coli* and the four organisms studied. This supports the notion that many strategies for the regulation of sets of functions by organisms exist. Second, despite variability among the ARs in different organisms, some ARs are well conserved over both short and long phylogenetic distances. Nine ARs were identified as conserved above a level of 75% of shared genes among *E. coli* and three of the organisms. Two of these were absolutely conserved with respect to gene membership; both were related to respiratory functions and each of the organisms are members of the Gamma Proteobacteria. Conservation was also identified for *E. coli, S. oneidensis*, and *S. aureus*, for two ARs associated with tryptophan amino acid biosynthesis and transport of phosphate. Third, having different levels of expression data for each organism has an impact on the quality of the ARs inferred. This can be seen as fewer genes being included in ARs and a higher proportion of genes being grouped into the static ARs.

We are continuing to investigate more statistically rigorous ON/OFF calling algorithms, which, instead of dichotomizing gene calls, yield a confidence metric that the gene is ON/OFF in a given condition. A calibrated continuous approach to ON/OFF calling may allow for more robustness in the calls. As new technologies are developed, and as current next generation sequencing technologies become more widely used and affordable, expression data for more experimental conditions will become available. This will lead to an improvement of the inference of ARs for the organisms considered in this study and any organism for which sufficient expression data are produced.

While improvements in the AR inference algorithm can be made, the current implementation of the method has been successfully used to extend our knowledge of the *B. subtilis* regulatory network (Faria et al., 2016). In that work, the atomic regulon inference approach was used to propose new additions to the *B. subtilis* regulatory network and led to the proposal of new functional annotations for genes. We integrated the ARs computed for this study into the SEED, allowing users to see the co-regulation of all genes in the ARs. These data complement the set of comparative genomics tools available in the SEED system. This information can be exceptionally valuable for researchers and curators, as co-regulation can group genes with well-known annotated functions with hypothetical genes, providing clues for new functional annotations. The AR inference algorithm and underlying gene ON/OFF calling algorithm have been included as a tool in KBase (http://www.kbase.us), which allows users to analyze their expression data in a feature rich platform designed to aid the discovery of new biological insights through the integration of multiple data types. We anticipate that a readily accessible implementation for the calculation of atomic regulons will enable many researchers to explore the relationships among these fundamental units of cellular function not only within their organism of choice, but also across the bacterial domain.

## Author contributions

RO, RDO, JD, MD, AB, NT, and JF developed the new atomic regulon inference algorithm. RO, RDO, and MD wrote the code for computing atomic regulons from expression data. RO and RDO developed the website for viewing atomic regulons. RO, JF, PW, and AB conducted comparative analysis of regulons. RT computed the CLR support for all atomic regulons from all methods. PW and JF applied hierarchical and k-means clustering methods to compute regulons. JD, PW, and JF ran sensitivity analysis on all methods. JF, JE, and BP developed the implementation of the algorithm in the DOE Systems Biology Knowledgebase (KBase). JF and CH primarily wrote the manuscript, with contributions and editing from all authors. CH, RO, MD, AB, RS, NT, IR, and MR conceived of the project and oversaw work.

## Funding

JF acknowledges funding from [FRH/BD/70824/2010] of the FCT (Portuguese Foundation for Science and Technology) PhD program. CH and PW were supported by the National Science Foundation under grant number EFRI-MIKS-1137089. RT was supported by the Genomic Science Program (GSP), Office of Biological and Environmental Research (OBER), U.S. Department of Energy (DOE), and his work is a contribution of the Pacific Northwest National Laboratory (PNNL) Foundational Scientific Focus Area. This work was partially supported by an award from the National Science Foundation to MD, AB, NT, and RO (NSF ABI-0850546). This work was also supported by the United States National Institute of Allergy and Infectious Diseases, National Institutes of Health, Department of Health and Human Service [Contract No. HHSN272201400027C].

### Conflict of interest statement

The authors declare that the research was conducted in the absence of any commercial or financial relationships that could be construed as a potential conflict of interest. The reviewer AP and handling Editor declared their shared affiliation, and the handling Editor states that the process nevertheless met the standards of a fair and objective review.

## Supplementary material

Supplementary Table S1 **1. 80 functional roles considered to be “always ON.”**.

Supplementary Table S2 **Complete set of atomic regulons computed for *E. coli***.

Supplementary Table S3 **Results from comparative analysis**.

CLR Supplementary Table CS-1 **CLR Support for Atomic Regulons (ARs)**. Method: Percentage of support for an AR is given by the percent of genes in the AR that have at least one high-scoring CLR edge that links to another gene in the same AR.

CLR Supplementary Table CS-2A **Support for our Atomic Regulons (ARs)**. Method: Percentage of support for an AR is given by the percent of all possible gene-to-gene connections in the same AR that have a corresponding high-scoring (above > SD4) CLR edge. This is a much tougher support criterion to meet than the criterion used in CLR Supplementary Table CS-1.

CLR Supplementary Table CS-2B **Support for k-means clusters**. Method: Percentage of support for an AR (k-means cluster) is given by the percent of all possible gene-to-gene connections in the same cluster that have a corresponding high-scoring (above > SD4) CLR edge.

CLR Supplementary Table CS-2C **Support for hierarchical clusters**. Method: Percentage of support for an AR (hierarchical cluster) is given by the percent of all possible gene-to-gene connections in the same cluster that have a corresponding high-scoring (above > SD4) CLR edge.
