# Supplementary material for: Computing and Applying Atomic Regulons to Understand Gene Expression and Regulation
Source: Front Microbiol. 2016 Nov 24;7:1819. doi: 10.3389/fmicb.2016.01819 (PMC5121216; doi:10.3389/fmicb.2016.01819)
Supplement: CLR Supplementary Table CS-1 — CLR Support for Atomic Regulons (ARs). Method: Percentage of support for an AR is given by the percent of genes in the AR that have at least one high-scoring CLR edge that links to another gene in the same AR. [file DataSheet3.docx]

***Supplement on CLR Validation***

***Validation of ARs by testing consistency with gene-to-gene interactions predicted by CLR***

The CLR algorithm, a mutual information-based method for regulatory network inference from gene expression data (Faith et al, 2007), was used on the same set of expression data that was used to build our ARs for *E. coli* (907 expression values for each of 4,329 *E. coli* genes). The CLR calculations were done on the DeGNServer web site, where the CLR algorithm has been parallelized to handle large gene sets (Li et al., 2013). A CLR score was recorded for each possible unique gene pair (each possible gene-to-gene connection). We thus obtained a set of 9,367,956 CLR scores [(4329 * 4328) / 2] for all unique gene pairs. We used the highest-scoring subset of CLR gene-to-gene edges to validate our atomic regulons. In doing this, two questions arose: (1) where do we (empirically) set the lower score bound on the CLR-derived gene-to-gene set to use for such validation, and (2) how exactly do we measure CLR support of the atomic regulons?

In regard to question (1), we note that the pair-wise CLR calculations are independent. Also, we assume that the average CLR score represents a background value reflective of non-regulation (non-correlation) between a gene pair, which is reasonable given that regulatory networks are sparse. (See Faith et al., 2007.) Thus, the distribution of CLR scores, if none of the genes are being regulated by or being co-regulated with other genes (our null hypothesis), would form a normal distribution about the average CLR score, with high (and low) scores deviating from the average by chance. Based on this, we can use known measures on normal distributions to establish the lower bound we want. That is, we calculated the average value of the nine million-odd CLR scores (0.606421) and the standard deviation (1.038146). We then also calculated a lower bound in terms of a CLR score that matches a given number of standard deviations (SDs) above the mean. For an SD of 4.0, the matching CLR score is the average + 4 SDs = 4.759. Is this a high enough CLR lower bound to ensure gene-to-gene connections that reflect biological co-expression, and not false positive connections seen by chance? We can estimate the reliability using the known shape of a normal distribution. At four SDs above the mean, only one CLR score in 31,574 is expected to meet or exceed that bound by chance, in the positive direction tail. That is, only 0.000032 (0.0032%) of the gene-to-gene pairs are expected to have a CLR score that high due to a false positive correlation in their gene expression patterns. Applying that to our set of 9,367,956 gene pairs, we find that 299.8 edges are expected to have scores that high. Thus, only 300 (0.42%) of the 71,678 gene-to-gene edges found by CLR to have scores >= 4.759 are expected by chance, and thus the SD4-bounded set should be a reliable subset to use to check the atomic regulons, and we use that subset here. This relatively small set is 0.765% (< 1%) of the total set of 9,367,956 possible edges for which CLR calculated scores.

In regard to question (2), we use the CLR edges in two ways to establish support: first, if a gene X in atomic regulon A is in one of the selected high-scoring CLR edges, and the other gene in that edge is also in regulon A, then we say that CLR supports gene X being included in regulon A, since CLR shows it being co-regulated with (or regulating, or being regulated by) at least one other gene in the same regulon. Individual gene support is then totaled to the regulon level, which we show in CLR Supplementary Table CS-1.

**CLR Supplementary Table CS-1 - CLR Support for Atomic Regulons (ARs). Method: percentage of support for an AR is given by the percent of genes in the AR that have at least one high-scoring CLR edge that links to another gene in the same AR.**

|  | **All 646 ARs** | **ARs having 3 or more genes** | **ARs having 4 or more genes** | **ARs having 13 or more genes** |
| --- | --- | --- | --- | --- |
| % of CLR support | # of ARs | # of ARs | # of ARs | # of ARs |
| 0 % | 75 | 9 | 1 | 0 |
| ]0%, 40%] | 2 | 2 | 2 | 0 |
| ]40%, 60%] | 8 | 8 | 8 | 0 |
| ]60% , 80%] | 37 | 36 | 14 | 0 |
| ]80%, 100%[ | 12 | 12 | 12 | 5 |
| 100% | 513 | 251 | 146 | 10 |
| Total # of ARs | 646 | 318 | 183 | 15 |

For example, if 7 of the 10 genes in regulon A are found by CLR to have high-scoring edges to at least one other gene in regulon A, then we say that regulon A has 70% support by CLR and we add one count in our tables to the number of regulons falling in the range of 60% to 80% support. CLR Supplementary Table CS-1 shows the number of regulons that fall into a given range of support for each of the full set of 646 *E. coli* regulons (spanning 2603 genes combined). The table is broken out into percentage ranges according to their level of CLR support.

The results in CLR Supplementary Table CS-1 show strong support for the atomic regulons. That is, 513 of all 646 regulons (79.4%) have 100% support, with only 75 regulons (11.6% of the total 646) having no support. When we restrict the regulons to the 318 that have three genes or more, 251 of 318 regulons (78.9%) have 100% support and only 11 (3.46%) have <= 40% support. Similarly, ARs that have four or more genes show 100% CLR support for 146 ARs (79.8% of the 183 total). The subset of larger ARs (with 13 or more genes) have similar good results, with all 15 such regulons showing >= 80% support.

We can also impose a tougher criterion to gauge CLR support: we can ask what percentage of *all possible* edges between the genes in a regulon are also found in our high-scoring subset of CLR-derived edges. We have done so, and the results are shown in CLR Supplementary Table CS-2. For example, if a regulon has six genes, then (6*5)/2 = 15 unique pair-wise edges can be formed within that gene set. If CLR found 10 edges that used only genes from that regulon (using no outside genes in the pair-wise edges), then we would state, according to this second criterion, that CLR gives that regulon 10/15 = 66.7% support.

Again, the results show strong support for the atomic regulons. 403 of all 646 regulons (62.4%) in CLR Supplementary Table CS-2A have 100% support, with only 75 regulons (no increase from CLR Supplementary Table CS-1) having zero support. When we restrict the regulons to the 318 that have three genes or more, 198 regulons (44.3%) have 100% support, 227 (71.4%) have >= 60% support, and 63 (19.8% of the 318) have < 40% support. ARs that have four or more genes have 100% support for 70 (38.5% of the 183), have >= 80% support for 97 (53.0%), with only 35 regulons (19.1%) having < 40% support. In the set of larger ARs (with 13 or more genes), 6 of the 15 ARs show >= 80% support. with 5 (33.3%) showing <= 40% support. Considering the size of the possible set of edges for larger-size regulons (for example, 78 possible edges for a regulon of 13 genes), CLR is providing an independent test on the gene expression data that is supporting even the largest regulon gene sets.

For comparison, we also computed CLR support of two sets of clustered genes, with the genes clustered based on similarity of gene expression, comparing against cluster sets created by the k-means and hierarchical clustering methods, as described earlier in this article. The size of the two clustering sets obtained via hierarchical clustering and k-means clustering were set to roughly match the level of granularity as is present in the AR regulon set. As described in the main text, the clustering algorithm runs were adjusted so that the number of clusters produced closely matched the number (646) of our regulons. We then evaluated CLR support of all the possible edges in each cluster, exactly as we did for the regulons, using the same subset of high-scoring CLR-based gene-to-gene-connections. The results are shown in CLR Supplementary Tables CS-3B and CS-3C. The k-means clusters show far worse CLR support than our regulons (CLR Supplementary Table CS-3B). For example, 66.7% of all 646 regulons have > 80% support, while only 28.7% of the k-means clusters (i.e., 155 the 540 non-singleton k-means clusters) achieve that support level. Also, at the AR level of granularity, the 645 clusters produced by hierarchical clustering produced 390 gene singletons, leaving only 255 useful clusters with two or more genes that could be evaluated against CLR results. In that reduced set of clusters containing two genes or more, the hierarchical clusters did much better in terms of CLR support than the k-means clusters, though still not matching the support shown by CLR for the regulons (CLR Supplementary Table CS-3C). For example, while the (reduced) set of 255 hierarchical clusters shows, as a whole, a slightly better result at the 80% support level – 69.4% of the 255 clusters reach that level, as compared to 66.7% of the 646 regulons - as soon as the number of genes in a cluster increases to more than two, our AR set shows more support. At the 80% support level and a regulon/cluster size of three genes or more, the regulons have slightly more support. At four genes or more, the difference grows to 53.0% vs. 43.2%, and by the time we reach large hierarchical clusters and regulons (13 or more genes), the difference in favor of the regulon set grows to 40% vs. 16%. And in every category (see CLR Supplementary Table CS-3C), the regulon set clearly beats the hierarchical clustering set in terms of having a smaller percentage with small (< 40%) CLR support, with the difference again growing dramatically as the cluster size increases from the minimum size of two. In summary: our new method of constructing atomic regulons do best, showing the most support.

**CLR Supplementary Table CS-2A - Support for our Atomic Regulons (ARs). Method: percentage of support for an AR is given by the percent of all possible gene-to-gene connections in the same AR that have a corresponding high-scoring (above > SD4) CLR edge. This is a much tougher support criterion to meet than the criterion used in CLR Supplementary Table CS-1.**

|  | **All 646 ARs** | **ARs having 3 or more genes** | **ARs having 4 or more genes** | **ARs having 13 or more genes** |
| --- | --- | --- | --- | --- |
| % of CLR support | # of ARs | # of ARs | # of ARs | # of ARs |
| 0 % | 75 | 9 | 1 | 0 |
| ]0% , 40%] | 56 | 56 | 34 | 5 |
| ]40% , 60%] | 26 | 26 | 26 | 3 |
| ]60% , 80%] | 59 | 59 | 25 | 1 |
| ]80%,100%[ | 27 | 27 | 27 | 4 |
| 100% | 403 | 141 | 70 | 2 |
| Total # of ARs | 646 | 318 | 183 | 15 |

**CLR Supplementary Table CS-2B - Support for k-means clusters. Method: percentage of support for an AR (k-means cluster) is given by the percent of all possible gene-to-gene connections in the same cluster that have a corresponding high-scoring (above > SD4) CLR edge.**

|  | **All 540 non-singleton Clusters** | **Clusters having 3 or more genes** | **Clusters having 4 or more genes** | **Clusters having 13 or more genes** |
| --- | --- | --- | --- | --- |
| % CLR support | # of Clusters | # # of Clusters | ## of Clusters | # # of Clusters |
| 0 % | 68 | 47 | 29 | 2 |
| ]0% , 40%] | 255 | 255 | 205 | 88 |
| ]40% , 60%] | 30 | 30 | 30 | 2 |
| ]60% , 80%] | 32 | 32 | 17 | 1 |
| ]80%,100%[ | 20 | 20 | 20 | 1 |
| 100% | 135 | 88 | 48 | 0 |
| Total | 540 | 472 | 382 | 94 |

**CLR Supplementary Table CS-2C - Support for hierarchical clusters – Method: percentage of support for an AR (hierarchical cluster) is given by the percent of all possible gene-to-gene connections in the same cluster that have a corresponding high-scoring (above > SD4) CLR edge.**

|  | **All 255 non-singleton Clusters** | **Clusters having 3 or more genes** | **Clusters having 4 or more genes** | **Clusters having 13 or more genes** |
| --- | --- | --- | --- | --- |
| % CLR support | # of Clusters | # # of Clusters | # # of Clusters | ## of Clusters |
| 0 % | 13 | 6 | 2 | 0 |
| ]0% , 40%] | 43 | 43 | 35 | 19 |
| ]40% , 60%] | 7 | 7 | 7 | 1 |
| ]60% , 80%] | 15 | 15 | 10 | 1 |
| ]80%,100%[ | 8 | 8 | 8 | 3 |
| 100% | 169 | 71 | 33 | 1 |

| Total | 255 | 150 | 95 | 25 |
| --- | --- | --- | --- | --- |

Hence, we have demonstrated using CLR that there is strong support from an independent information-theoretic method for most of the coexpression inferred in the ARs computed by our approach, and that this support is higher for the ARs predicted by our approach compared to those predicted by competing approaches.

NOTE: Figures 4a and 4b in the main text showing CLR support results are based on data compiled in the CLR Supplementary Tables CS-2A, CS-2B, and CS-2C shown above.
