# Supplementary material for: Computing and Applying Atomic Regulons to Understand Gene Expression and Regulation
Source: Front Microbiol. 2016 Nov 24;7:1819. doi: 10.3389/fmicb.2016.01819 (PMC5121216; doi:10.3389/fmicb.2016.01819)
Supplement: Supplementary file 4 [file DataSheet4.pdf]

How to use “Compute Atomic Regulons” in the DOE Knowledgebase of Systems Biology (KBase)

1st - Create a KBase Account

- Sign up for a KBase account at: http://kbase.us/sign-up-for-a-kbase-account/

2nd – Sign in the KBase Narrative Interface

- Access the Kbase Narrative Interface at: https://narrative.kbase.us/
- Create a “New Narrative”.

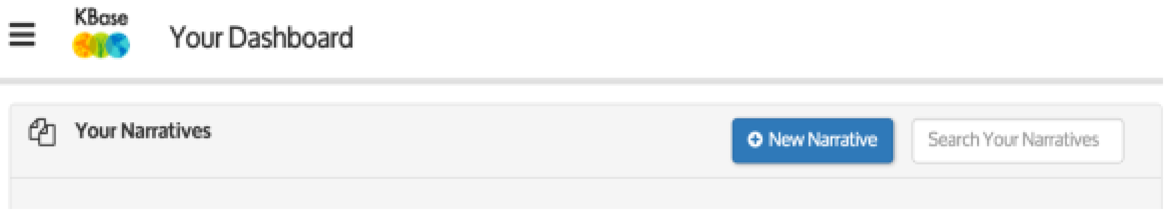

3rd – Load data in the Narrative

- Computation of Atomic Regulons requires a “Genome” and “Expression Dataset/Matrix”.
- Please see the following tutorial to load an “Expression Matrix” on KBase: https://kbase.us/data-upload-download-guide/expression-matrix/
- Please see the following tutorial to load a “Genome” on KBase: https://kbase.us/data-upload-download-guide/genome/
- Genomes can also be add from the vast collection of KBase Public genomes, by hitting “+” in the “DATA” panel.

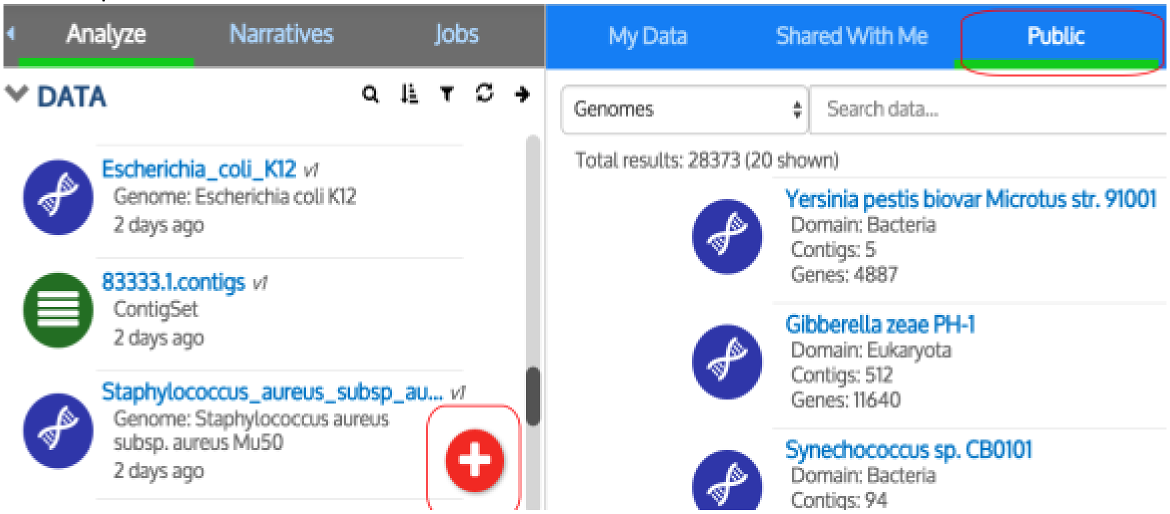

ALTERNATIVELY

- All genomes and expression datasets used in the manuscript are available at in the following Public Narrative: https://narrative.kbase.us/narrative/ws.14533.obj.1
- To use the data and edit the narrative contents, create your own copy of the public narrative by hitting "Copy" in the top right corner.

4th – Toggle between Release and Beta Versions

- If the "Compute Atomic Regulons" method is not available in the Release version ("R") of the "APPS & METHODS" catalog, please search for Beta version. Hit "B" as shown below in "APPS & METHODS" to toggle between Release and Beta versions.
- In the search bar, search for "Compute Atomic Regulons".

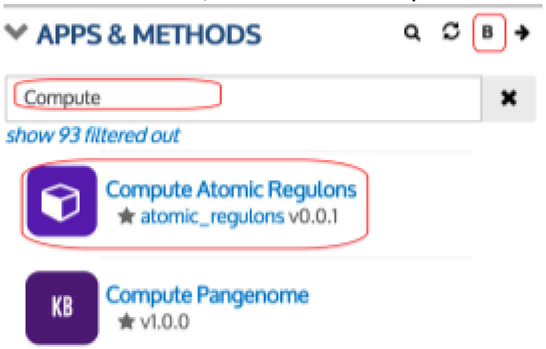

5th – Compute Atomic Regulons

- Add the "Compute Atomic Regulons" method the Narrative.
- Select "Genome" and "Expression Data Set" of interest.
- "Expression Threshold" sets the Pearson Correlation Coefficient (PCC) cut-off used to split operon and subsystem based clusters.

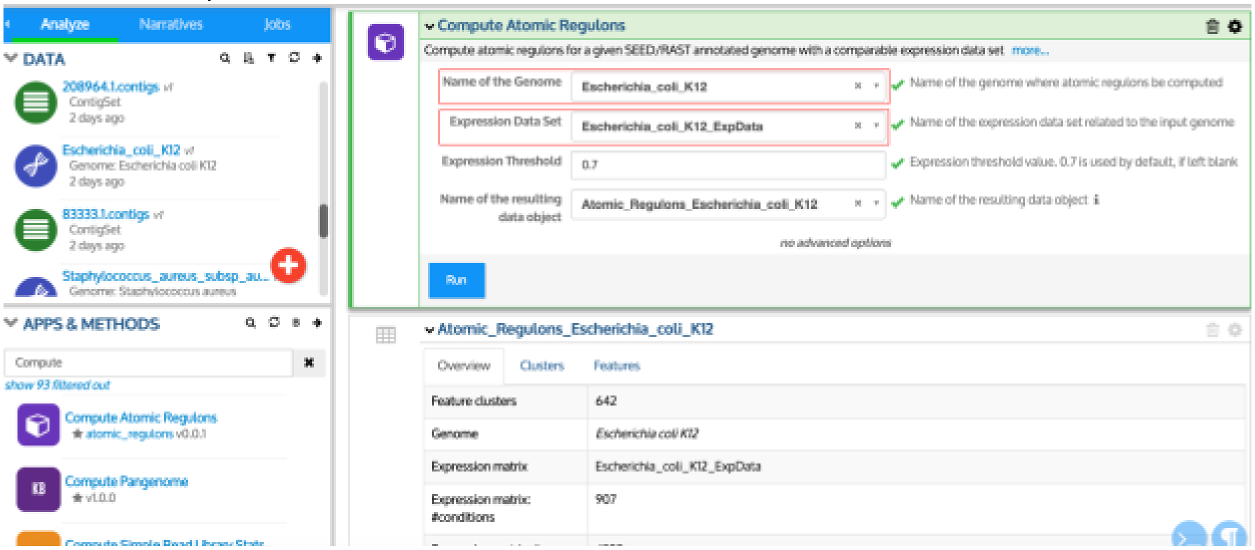

Office of Biological and Environmental Research

U.S. DEPARTMENT OF ENERGY
Office of Science

KBase
PREDICTIVE BIOLOGY
DOE Systems Biology Knowledgebase
